# CCR7 Is Recruited to the Immunological Synapse, Acts as Co-stimulatory Molecule and Drives LFA-1 Clustering for Efficient T Cell Adhesion Through ZAP70

**DOI:** 10.3389/fimmu.2018.03115

**Published:** 2019-01-14

**Authors:** Julia M. Laufer, Ilona Kindinger, Marc Artinger, Andreas Pauli, Daniel F. Legler

**Affiliations:** ^1^Biotechnology Institute Thurgau (BITg) at the University of Konstanz, Kreuzlingen, Switzerland; ^2^Konstanz Research School Chemical Biology, Department of Biology, University of Konstanz, Konstanz, Germany; ^3^Graduate School for Cellular and Biomedical Sciences, University of Bern, Bern, Switzerland; ^4^Klinikum Konstanz, Konstanz, Germany

**Keywords:** CCR7, chemokine receptor, ZAP70, TCR, LFA-1, integrins, immunological synapse

## Abstract

The chemokine receptor CCR7 guides T cells and dendritic cells to and within lymph nodes to launch the onset of adaptive immunity. Here, we demonstrate that CCR7 in addition acts as a potent co-stimulatory molecule in T cell activation. We found that antigen recognition and engagement of the TCR results in CCR7 accumulation at the immunological synapse where CCR7 and the TCR co-localize within sub-synaptic vesicles. We demonstrate that CCR7 triggering alone is sufficient to recruit and activate ZAP70, a critical kinase for T cell activation, through Src kinase, whereas TCR CCR7 co-stimulation results in increased and prolonged ZAP70 kinase activity. Finally, we show that ZAP70, acting as adapter molecule, is critical for CCR7-mediated inside-out signaling to integrins, thereby modulating LFA-1 valency regulation to promote cell adhesion, a key step in immunological synapse formation and efficient T cell activation.

## Introduction

Efficient adaptive immunity necessitates the encounter of pathogen-derived antigens presented by dendritic cells (DCs) and antigen-specific T cells within lymph nodes. The chemokine receptor CCR7 and its ligands CCL19 and CCL21 are vital for the induction of adaptive immune responses by guiding antigen-bearing DCs as well as naïve and memory T cells from peripheral tissues to lymph nodes and by controlling the intranodal motility and scanning behavior of T cells (1, 2). The integrin lymphocyte function-associated antigen 1 (LFA-1) regulates the arrest of T cells on high endothelial venules (HEVs) to allow emigration into lymph nodes as well as their interaction with DCs forming the immunological synapse, permitting optimal T cell activation in the context of co-stimulatory molecules (1, 3–5). Notably, CCR7 signaling not only controls cell locomotion but also drives conformational changes of integrins promoting high affinity ligand binding required for cell arrest (6–8). Moreover, CCR7 contributes to T cell receptor (TCR) signaling and co-stimulation (9, 10). How CCR7 triggering feeds into distinct signaling pathways to tune cell adhesion and co-stimulation on the molecular level is still poorly understood.

T cells, particularly the naïve ones, recirculate via blood through secondary lymphoid organs, whereby homing is orchestrated by CCR7 signaling that induces clustering and high affinity conformation of LFA-1 allowing T cells to arrest on HEVs, to crawl on HEVs and to diapedese in a chemokine dependent manner (8, 11, 12). In lymph nodes, CCR7 signaling controls T cell motility and speed rather than directionality and arrest, and T cells migrate in a random biased walk along the network of fibroblastic reticular cells (FRCs) (2, 5). FRCs simultaneously expose immobilized CCL21 and LFA-1 ligands on their surface, thereby facilitating efficient T cell scanning for specific antigens presented by DCs (3, 13). Besides this, CCR7 triggering contributes to T cell activation, whereby reciprocal crosstalks between CCR7 and TCR have been described. On the one hand, TCR-engagement modulates responsiveness of T cells to CCR7 ligands via Src kinases (10). On the other hand, CCR7 signaling owns co-stimulatory functions in T cell priming in mice (9). The precise molecular mechanism and whether CCR7 acts as a co-stimulatory molecule also in human T cells remains to be determined. Nevertheless, efficient T cell activation necessitates the formation of a stable immunological synapse at the interface between the T cell and the DC (4). It is well-established that LFA-1 and the TCR on the T cell and the intracellular adhesion molecule-1 (ICAM-1) and the MHC-peptide complex on the DC define the immunological synapse. Using a model of superantigen-loaded B cells as antigen-presenting cell (APC), which do not expose CCL21 on their surface, did not reveal a role for CCR7 at the interface between T cells and the APC (14), questioning whether and how CCR7 can act as a co-stimulatory molecule. As DCs present immobilized CCL21 and ICAM-1 on their surface (3, 15) and because CCR7 triggering on T cells is key to induce clustering and affinity regulation of LFA-1 by inside-out signaling (6, 16), it is conceivable that CCR7-mediated inside-out signaling to LFA-1 may contribute to the formation of a stable immunological synapse resulting in efficient T cell co-stimulation.

We recently identified the non-receptor tyrosine kinase ζ-associated protein of 70 kDa (ZAP70) as direct interaction partner of chemokine-stimulated, tyrosine phosphorylated CCR7 (17). ZAP70 is one of the most-upstream components of TCR signaling and crucial for T cell development and function (18). In addition, ZAP70 constantly associates with LFA-1 and becomes phosphorylated by the Src kinase Lck upon ICAM-1 to LFA-1 binding, and inhibiting ZAP70 activity was found to interfere with LFA-1 outside-in signaling and LFA-1-dependent cell motility (19, 20). Moreover, CXCR4-induced ZAP70 activation results in phosphorylation of Vav1, a guanine nucleotide exchange factor for Rac/Rho GTPases, and its dissociation from talin enabling talin-integrin association and hence, inside-out signaling and activation of the integrin very late antigen-4 (VLA-4) in T cells (19–21). Remarkably, we recently identified ZAP70, which is aberrantly expressed in chronic lymphatic leukemia (CLL), to enhance chemokine-driven CLL arrest by promoting integrin clustering (22).

Herein, we found that CCR7 is recruited to the immunological synapse and acts as co-stimulatory molecule in human T cells. We discovered that CCR7 associates with the ζ chain of the TCR and recruits and activates the tyrosine kinase ZAP70 upon chemokine triggering. Co-stimulation of CCR7 and the TCR resulted in a prolonged and enhanced kinase activity of ZAP70. Interestingly, we found an additional role for ZAP70 as adapter protein in CCR7-driven inside-out signaling modulating LFA-1 valency regulation to promote cell adhesion, a determining step in the formation of an immunological synapse. Consistent with this, we found that co-stimulation of CCR7 and the TCR resulted in enhanced CD69 induction and IL-2 secretion, hallmarks of efficient T cell priming.

## Materials and Methods

### Materials

Recombinant human CCL19 and CCL21 were purchased from PeproTech (Rocky Hill, NJ, USA). The following antibodies were used: PE-labeled anti CD69 (clone FN50) (Bio-Rad, Hercules, CA, USA), PE-labeled anti-IL-2 (BD Pharmigen, Franklin Lakes, NJ, USA), anti-human CCR7 used for immune fluorescence (SAB4500329) (Sigma-Aldrich, St. Louis, MO, USA), anti-human CCR7 (LifeSpan Biosciences, Seattle, WA, USA) used for PLA, anti-human Vav1 (9C1) (Abnova, Taipei City, Taiwan) used for PLA, anti-human CCR7 APC (FAB197A) (R&D Systems, Minneapolis, MN, USA), anti-humanCD3ζ (ab188850) (abcam, Cambridge, United Kingdom), anti-CD3 (ab5690) (abcam), anti-CD3 (clone OKT3; Janssen-Cilag, Beerse, Belgium), anti-ZAP70 (D1C10E) XP® Rabbit mAb (Cell Signaling, Danvers, MA), anti-ZAP70 (phospho Y319) antibody (ab131270) (abcam), anti-YFP1 (E385) (abcam), anti-YFP2 (11814460001) (Roche, Basel, Switzerland), monoclonal anti-HA-HRP (clone HA7) (Sigma-Aldrich), anti-HA antibody (clone HA7) (Sigma-Aldrich), anti-CD11a antibody [EP1285Y] (ab52895) (abcam), anti-CD18 antibody [MEM-48] (ab657) (abcam), mAb24 anti-CD11+CD18 antibody [24] (ab13219) (abcam). Phalloidin coupled to Alexa647 was purchased from Life Technologies (Carlsbad, CA, USA). PP2 and PTx were purchased from Merck-Millipore (Burlington, MA, USA). Piceatannol was from Sigma. HEL_34−45_ (ISQAVHAAHAEINEARG) and OVA_323−339_ (ISQAVHAAHAEINEARG) were purchased from JPT (Berlin, Germany).

### Construction of Expression Plasmids

Cloning of pcDNA3-CCR7-HA and pcDNA3-CCR7-EYFP has been described previously (23). The constructs pcDNA3-CCR7-YFP1/YFP2 were subcloned by PCR using full length CCR7 as template and specific primers (5′-CTG CGA ATT CAT GGA CCT GGG GAA ACC AAT G and 5′-CTA TAT CGA TTG GGG AGA AGG TGG TGG TG) into YFP-BiFC vectors (24). Analogously, the previously described CCR7-DNY (25) and CCR7-Y155F (17) mutants were cloned into BiFC vectors using the same strategy. The cloning of pcDNA3-CCR7-V317I-YFP1 and pcDNA3-CCR7-A315G-YFP1 was previously described (17). pDONR223-ZAP70 (26) was a gift from William Hahn & David Root (Addgene plasmid # 23887). The construction of pcDNA3-Vav1-YFP2, pcDNA3-ZAP70-YFP1/YFP2, pcDNA3-CD3ζ-YFP2 were performed analogously using following primer pairs: Vav1 (5′ CTG CAA GCT TCT TGT AGA AGC GCG TAT G 3′ and 5′ GCC GAT CGA TGC AGT ATT CAG AAT AAT CTT CC 3′); ZAP70 (5′CTT GGA ATT CAT GCC AGA CCC CGC GGC GCA C 3′ and 5′ GAA TAT CGA TGG CAC AGG CAG CCT CAG CCT T 3′); CD3ζ (5′ GAA CGA ATT CTT CTG CCT CCC AGC CTC TTT CT 3′ and 5′ GAA CAT CGA TTC AGG CCT TCC TGA GGG TTC TT 3′); and subcloned into the previously published N-terminally tagged YFP-BiFC vectors (24). Site directed mutagenesis was performed using the QuickChange II site directed mutagenesis kit (Agilent, Santa Clara, CA, USA) following manufacturer‘s instructions using the primers: for ZAP70-Y315F (5′ CCC ATG GAC ACG AGC GTG TTT GAG AGC CCC 3′ and 5′GGG GCT CTC AAA CAC GCT CGT GTC CAT GGG 3′) and for ZAP70-K369R (5′GCT TCA GCA CCC TGA TGG CCA CGT CGA TCT GC 3′ and 5′GCA GAT CGA CGT GGC CAT CAG GGT GCT GAA GC 3′). Cloning of pcDNA3-ZAP70-EGFP was done by performing PCR on full-length human ZAP70 using the primer pairs (5′ GAT AGA ATT CAT GCC AGA CCC CGC GGC GCA CC 3′ and 5′ CTT AGC GGC CGC GGC TGA TCA GCG AGC TCT AGC A 3′) and subsequently cloned into pcDNA3-EGFP. All primers were custom made by Microsynth (Balgach, Switzerland). pcDNA3.1 + ROZA XL and pcDNA3.1 + ROZA XL YF (27) were a kind gift of Annemarie Lellouch (Addgene plasmids #64194 and #64195).

### Isolation of Primary Human and Murine Cells, Cell Lines, and Transfection

Blood donation for research purposes was approved by the local ethics committee and individual donors gave written consent. PBMCs from healthy donors were enriched by density gradient centrifugation on Ficoll-Paque Plus (Amersham Biosciences, Little Chalfont, UK). Monocytes were separated from PBLs using anti-CD14-conjugated microbeads (Miltenyi, Gladbach, Germany). PBLs or CD3^+^ sorted (Miltenyi) T cells were cultured in RPMI-1640-medium (Gibco, Waltham, MA, USA) supplemented with 2% human AB serum (Lonza, Basel, Switzerland). Murine BMDCs were generated from bone-marrow of BALB/c mice. Cells were differentiated in RPMI-1640-medium supplemented with 2mM L-Glutamine (Gibco), 1% pen/strep (Lonza), 10% heat-inactivated FCS (Gibco), and 0.05% β-mercaptoethanol in the presence of 20 ng/ml GM-CSF for 8 days to obtain immature BMDCs. BMDCs were matured by addition of 100 ng/ml LPS for 24 h. HEK293 cells and stable HEK293 CCR7-HA or CXCR4-HA transfectants were grown and maintained in DMEM containing 10% FCS (Lonza) and 1% pen/strep (Lonza). HEK293 cells were transiently transfected using TransIT-LT1 (MirusBio, Madison, WI, USA), according to the manufacturer's protocol. Mouse embryonic fibroblasts (MEFs) derived from wild-type or β-arrestin 1 and 2 double deficient animals were grown and maintained in DMEM containing 10% FCS (Lonza) and 1% pen/strep (Lonza). MEFs were transiently transfected by Lipofectamin 3,000 (Life technologies), according to the manufacturer‘s protocol. The murine T cell hybridoma 3B11 and murine B cell lymphoma LK35.2 (28) were provided by Günter J. Hämmerling. The DO11.10 T hybridoma cell line was a kind gift of Thomi Brunner. DO11.10, 3B11, and LK35.2 cells were grown and maintained in RPMI-1640-medium containing 10% FCS (Lonza), 1% of non-essential amino acids (Lonza), and 1% pen/strep (Lonza). 3B11 and DO11.10 stably transfected with pcDNA3-CCR7-EYFP were generated using electroporation and selection with G418 (Gibco). ZAP70-deficient Jurkat P116 were kindly provided by Margot Thome-Miazza. Jurkat cells were grown and maintained in RPMI-1640-medium containing 10% FCS (Lonza), 1% of non-essential amino acids (Lonza) and 1% pen/strep (Lonza). Jurkat P116 cells stably expressing ZAP70-EGFP were generated by transfection with Lipofectamin3000 (Life Technologies) and selection with G418 (Gibco). Jurkat P116 cells were transiently transfected using the Neon Transfection system (Thermo Fisher Scientific, Waltham, MA, USA).

### Bimolecular Fluorescence Complementation (BiFC) Assay

BiFC was determined mainly in HEK293 cells transiently transfected in a 1:1 ratio with various splitYFP1 and splitYFP2-tagged constructs. BiFC between ZAP70/ZAP70-Y315F-YFP1 and Vav1-YFP2 was performed in HEK293 cells stably expressing either CCR7-HA or CCR7-DNY-HA. Where indicated BiFC was conducted in MEFs derived either from wild-type mice or from β-arrestin 1/2 double deficient mice. One hundred and fifty thousand cells per well were seeded into 6-well plates containing coverslips for microscopy or without coverslips for flow cytometry. 24 h later cells were transfected as described above. The subsequent day, transfected cells were left untreated or were pre-treated with 100 ng/ml PTx for 4 h or 10 μM PP2 for 4 h. Cells were stimulated or not with 0.5 μg/ml CCL19 or CCL21, washed and fixed in 4% formaldehyde for 10 min at room temperature. For flow cytometry analysis, cells were subsequently detached, filtered (50 μm filter), and YFP fluorescence was analyzed on a LSRII flow cytometer (BD Biosciences). For immunofluorescence microscopy, cells on coverslips were permeabilized using 0.2% Triton X-100 and 0.125% SDS in PBG (20 mM glycine and 3% BSA in PBS pH 7.4) and incubated with the appropriate primary antibody in PBG, followed by incubation with AlexaFluor-labeled secondary antibodies (Life Technologies). Coverslips were mounted using polyvinyl alcohol mounting medium with DABCO (Sigma-Aldrich). Confocal images were acquired on a Leica TCS SP5 II laser scanning microscope using a 63x/1.4 NA oil-immersion objective (Leica, Wetzlar, Germany).

### Proximity Ligation Assay (PLA)

For analysis of the interaction of endogenous CCR7 with CD3ζ, CCR7 with ZAP70 and ZAP70 with Vav1 human primary PBLs were placed on poly-L-lysine coated coverslips, stimulated with 0.5 μg/ml CCL19 or CCL21 for the indicated time-points or left unstimulated. Subsequently, cells were fixed in 4% formaldehyde, blocked in Fc-block for 2 h, permeabilized, washed and incubated in primary antibody diluted 1:50 in Fc-block for 2 h. Slides were washed and PLA was performed using the reagents from the Duolink® proximity ligation assay (Sigma-Aldrich) as described (17). Secondary PLA antibodies harboring short nucleotide sequences diluted 1:5 in antibody diluent were added for 1 h at 37°C. Oligonucleotides were ligated at 37°C and rolling circle PCR with fluorescent nucleotides was performed for 2 h at 37°C. Slides were washed, nuclei stained with Hoechst and mounted. PLA was visualized on an inverted Zeiss Axiovert200 microscope. Quantification of PLA signals was performed in ImageJ.

### Förster Resonance Energy Transfer (FRET) Measurements

Jurkat cells were transiently transfected with pcDNA3.1+ROZA-XL or pcDNA3.1+ROZA-XL-YF using the Neon® transfection system (Thermo Fisher Scientific). Twenty-four hours after transfection cells were placed on poly-L-lysine coated coverslips and stimulated with 0.5 μg/ml CCL19/CCL21 or 5 μg/ml anti-CD3 (clone OKT3) or co-stimulation with chemokine and anti-CD3 antibody. Subsequently, cells were fixed in 4% PFA. Coverslips were mounted using polyvinyl alcohol mounting medium with DABCO (Sigma-Aldrich). FRET efficiency of the ROAZA-XL FRET sensors was measured on a Leica TCS SP5 II confocal microscope by donor-recovery after acceptor photo-bleaching (AB) using the corresponding FRET-wizard of the Leica LSM software. Briefly, a pre-bleaching image was taken, acceptor of the whole cell was bleached at 100% laser intensity and FRET efficiency was calculated for each pixel with FRET_eff_ = (D_postbleach_ – D_prebleach_)/D_postbleach_ using the macro of the FRET-AB wizard.

### Immune Synapse Formation Assay

LK35.2 APCs or mature murine BMDCs were loaded with 10 μM HEL_34−45_ or 10 μM OVA_323−339_ antigen for 1 h, washed and placed on poly-L-lysine coated coverslips. 30 min later 3B11 or DO11.10 T cells stably expressing CCR7-YFP were added. Cells were left unstimulated or stimulated with 0.5 μg/ml CCL19 or CCL21 for 1 h. Cells were fixed in 4% PFA for 10 min at room temperature. For staining, cells were permeabilized and incubated with the appropriate primary antibody, followed by incubation with AlexaFluor-labeled secondary antibodies (Life Technologies). Coverslips were mounted using polyvinyl alcohol mounting medium with DABCO (Sigma-Aldrich). Confocal mages were acquired on a Leica TCS SP5 II laser scanning microscope using a 63x/1.4 NA oil-immersion objective (Leica). 3D reconstitution was performed by Huygens software using confocal images with z-step sizes of 0.08 μm. Quantification of co-localization was done by calculating the Pearson's correlation coefficient using ImageJ.

### Preparation of Cell Lysates

1 × 10^6^ Jurkat cells were lysed in NP-40 buffer [1%NP-40, 50 mM Tris-HCl pH 7,6, 150 mM NaCl, 2 mM EDTA, supplemented with proteinase inhibitor mix (Roche), pH 7.5] and total protein concentration was determined using 660 nm Protein Assay (Pierce, Waltham, MA, USA). Total protein amount was adjusted and cell lysates were transferred to SDS-PAGE gels and Western blot analysis was performed using indicated antibodies.

### Quantitative Real-Time PCR

Total RNA of T cells was isolated using the RNeasy Mini kit (Qiagen, Hilden, Germany) and transcribed into cDNA using random hexamer primers and the Hi Capacity cDNA Reverse Transcription kit (Applied Biosystems, Foster City, CA, USA). Amplification of transcripts was performed using the Fast SYBR Green PCR Master Mix on a 7900HT Fast Real-Time PCR System (Applied Biosystems) according manufacturer‘s instructions. For detection of IL-2 primer pairs were purchased from Qiagen. Data from real-time PCR were normalized to the mean of four different housekeeping genes (B_2_M, GAPDH, UBC, TBP). Relative mRNA expression was calculated with the 2^∧^−*delta* Ct method.

### Flow Cytometry Analysis of T Cell Activation

Human primary PBLs, isolated the day before as described above, were stimulated with graded concentrations of CCL19, CCL21 (0.01, 0.025, 0.1, 0.25, 0.5 μg/ml) or anti-CD3/anti-CD28 beads (T cell activation/expansion kit, Miltenyi Biotec) or co-stimulation with chemokine and anti-CD3/anti-CD28 beads. Twenty hours after stimulation, cells were fixed in 4% PFA for 10 min at room temperature and stained for CD69 using PE-labeled anti-CD69 (clone FN50, Bio-Rad) for 45 min at room temperature. Subsequently cells were washed and PE-fluorescence was measured. For analysis of IL-2 production, intracellular IL-2 staining was performed. For this, cells were treated with 10 μg/ml monensin for 5 h before fixation in 4% PFA. Cells were permeabilized using 0.1% saponin in PBS and 0.5% BSA. Intracellular staining was done in presence of 0.1% saponin using PE-labeled anti-IL-2 antibody (BD Biosciences) for 30 min at room temperature. Subsequently, cells were washed and fluorescence was analyzed on a LSRII flow cytometer (BD Biosciences). Quantification was done using FlowJo7 software.

### Flow Cytometry Analysis–mAb24 Staining

Jurkat P116 or Jurkat P116 ZAP70-GFP cells were stimulated with 0.5 μg/ml CCL19 or CCL21 in presence of 0.9 μg/ml mAb24 [anti-CD11a+CD18 antibody [24] (ab13219) (abcam)] for 10 min at 37°C in HBSS. Subsequently, cells were put on ice for 30 min, fixed with 4% PFA and stained with secondary goat-anti-mouse-IgG antibody coupled to Alexa647 (Life Technologies) in HBSS and 3% BSA. Cells were washed in HBSS and fluorescence was analyzed on a LSRII flow cytometer (BD Biosciences). Quantification was done using FlowJo7 software.

### Cell Migration Assay

LK35.2 APCs, stained with CellTracker^TM^ DeepRed (Thermo Fischer; 0.5 μM) were loaded with 10 μM HEL_34−45_ antigen for 1 h or left unloaded, washed and incubated with 3B11 T cells expressing CCR7-YFP in a 2:1 ratio for 15 min at 37°C. As a control, 3B11 CCR7-YFP T cells were incubated without LK35.2 APCs. Cells were transferred into the upper compartment of a 24-well Transwell® System with polycarbonate filters with a pore size of 5 μm (Corning Costar) and were allowed to migrate for 2 h to the lower compartment, containing chemokine-free medium or medium supplemented with CCL19 or CCL21 (0.5 μg/ml) as described (14). Cells that had migrated to the lower compartment were harvested and cell numbers were determined by flow cytometry on a LSR II flow cytometer (BD Biosciences).

### Immobilized ICAM-1 Adhesion Assay

Black 96-well plates with clear bottom (Costar) were pre-coated with 100 μg/ml protein A (Pierce) in PBS overnight at 4°C. Plates were washed in HBSS and coated with 10 μg/ml ICAM-1-Fc (R&D Biosystems) in HBSS for 1.5 h at 37°C. Subsequently, plates were washed and blocked for 1 h at 37°C using HBSS and 0.5% low-fat BSA (A7511, Sigma). Jurkat cells were stained with Vybrant® DiD cell labeling solution (Thermo Fisher Scientific) according to the manufacturer's protocol and stimulated with 0.5 μg/ml CCL19/CCL21 or 10 mM MgCl_2_ and let adhere to immobilized ICAM-1-Fc for 20 min in HBSS and 0.5% low-fat BSA. Non-adherent cells were washed off the plates by 3–4 washing steps and cell-associated fluorescence was measured using a Tecan® Spark 1 M microplate reader. Percentage of adherent cells was calculated in relation to unwashed wells (input).

### CD11a Cluster Analysis

Jurkat P116, Jurkat P116 ZAP70-GFP, or Jurkat P116 cells transiently transfected with ZAP70-K369R-GFP were placed on poly-L-lysine coated coverslips and left untreated or pre-treated with 50 μg/ml piceatannol or DMSO for 1 h and subsequently stimulated for 10 min with 0.5 μg/ml CCL19 or CCL21. Cells were fixed in 4% PFA and immunostaining for CD11a was done as described above, but omitting the permeabilization step. Confocal images were acquired on a Leica TCS SP5 II laser scanning microscope using a 63x/1.4 NA oil-immersion objective (Leica).

### Statistical Evaluation

Significant differences between groups were assessed using two-way ANOVA with Bonferroni post-test using GraphPad Prism 6. ^*^p < 0.05, ^**^ *p* < 0.01, ^***^p < 0.001, ^****^p < 0.0001.

## Results

### CCR7 Acts as a Co-stimulatory Molecule for Efficient Human T Cell Activation

**Figure 1:**
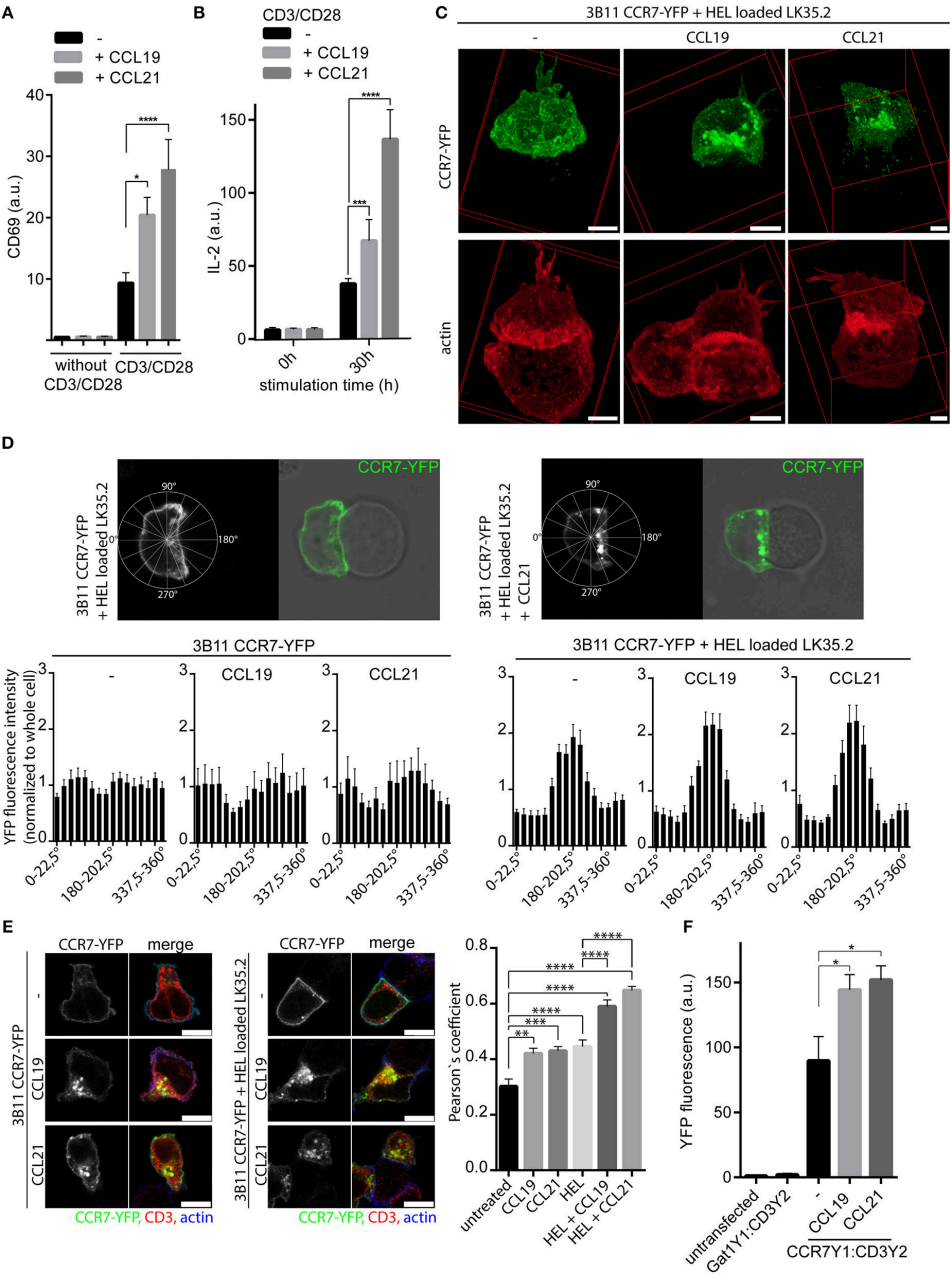
CCR7 acts as co-stimulatory molecule and is recruited to the immunological synapse**. (A)** Quantification of CD69 surface expression on human PBLs upon TCR stimulation using anti-CD3/CD28 coated beads and/or CCR7 stimulation (0.5 μg/ml CCL19 or CCL21) for 20 h measured by flow cytometry. Mean ± SEM of four individual donors. **(B)** Intracellular IL-2 staining in human PBLs upon TCR and/or CCR7 stimulation for 30 h measured by flow cytometry. Mean ± SEM of four individual donors. **(C)** Deconvoluted 3D reconstructions of CCR7-YFP in 3B11 T cells that were incubated for 1 h with HEL peptide-loaded LK35.2 APCs and simultaneously stimulated or not with 0.5 μg/ml CCL19 or CCL21. Scale bar, 3 μm. **(D)** Distribution and quantification of CCR7-YFP in 3B11 T cells conjugated or not to HEL peptide-loaded LK35.2 APCs and stimulated or not with chemokines (0.5 μg/ml for 1 h) in Rose plot sectors. Analysis of 30 cells derived from three independent experiments; scale bar, 3 μm. **(E)** Co-localization and quantification (Pearson‘s coefficient) between CCR7-YFP and CD3ζ in 3B11 T cells upon stimulation with chemokines (0.5 μg/ml for 1 h) alone or conjugated to HEL peptide-loaded LK35.2 APCs. Mean ± SEM of 15 cells derived from one out of three independent experiments; scale bars, 7.5 μm. **(F)** Flow cytometric quantification of CCR7-YFP1:CD3ζ-YFP2 interaction by BiFC in transfected HEK293 cells upon stimulation with 0.5 μg/ml CCL19 or CCL21 for 30 min. Gat1-YFP1:CD3ζ-YFP2 BiFC served as negative control. Mean ± SEM of three independent experiments. ^*^p < 0.05; ^**^p < 0.01; ^***^p < 0.001; ^****^p < 0.0001.

Whether the chemokine receptor CCR7 acts as co-stimulatory molecule in T cell activation is controversial as on one side CCL21 was recognized to mediate T helper cell co-stimulation in mice (9), whereas on the other side CCR7 was reported to be excluded from the immunological synapse of human T cells and superantigen-loaded EBV-B cells (14). We therefore first assessed the co-stimulatory capacity of CCR7 in primary human peripheral blood T cells (PBLs) in a molecular defined and controllable system using CCR7 ligands and anti-CD3/anti-CD28 antibodies coated to beads. PBLs were stimulated by CD3/CD28 triggering in the presence or absence of CCR7 ligands and T cell activation was determined by the induction of the early T cell activation marker CD69. TCR triggering alone induced surface expression of CD69, which was significantly higher if PBLs were co-stimulated with either of the two CCR7 ligands (Figure 1A, Supplementary Figures 1A,B). Similarly, TCR and CCR7 co-stimulated PBLs produced significantly more IL-2 compared to T cells stimulated solely via TCR (Figure 1B, Supplementary Figure 1C). Remarkably, T cells that were only exposed to CCL19 or CCL21 neither induced CD69 expression nor produced IL-2 (Figures 1A,B, Supplementary Figures 1A,C). The co-stimulatory effect gradually increase with higher chemokine concentrations and was more pronounced with CCL21 than with CCL19 in human T cells, which is consistent with a previous study in mice (9).

### TCR Engagement Results in CCR7 Accumulation at the Immunological Synapse

The co-stimulatory effect of CCL19 and CCL21 raises the question whether CCR7 localizes at the contact site between a T cell and the APC presenting the cognate antigen. To address this, we used the well-studied 3B11 T cells expressing a TCR specific for the hen egg lysozyme peptide HEL_34−45_ and LK35.2 B cells as APCs (28). We generated 3B11 T cells stably expressing CCR7-YFP and analyzed the localization of the chemokine receptor by confocal microscopy (Figures 1C,D). CCR7-YFP was evenly distributed at the entire plasma membrane and at some vesicular structures of 3B11 T cells in the absence of antigen-loaded APCs (Figure 1D). In contrast, loading of LK35.2 APCs with HEL antigens resulted in a profound redistribution and accumulation of CCR7-YFP at the immunological synapse (Figures 1C,D). Rose plot analysis on the CCR7-YFP distribution in 3B11 T cells revealed that CCR7 is enriched at the contact site only if the immunological synapse has been formed between 3B11 T cells and LK35.2 APCs loaded with the cognate peptide (Figure 1D). Our data on CCR7-YFP redistribution are in line with the study by Molon and colleagues showing that other chemokine receptors, namely CCR5-GFP and CXCR4-GFP, are recruited to the immunological synapse of antigen-loaded APCs (14), but distinct to the reported absence from the synapse of CCR7, which was indirectly stained by CCL21-Fc and a fluorescently labeled secondary antibody (14). Interestingly, in our study we observed that in the presence of chemokines CCR7-YFP mainly localized intracellularly at vesicular structures beneath the immunological synapse (Figures 1C,D). To corroborate the recruitment of CCR7 to the immunological synapse formed between DCs and T cells, we used murine bone-marrow-derived DCs (BMDCs) loaded with OVA_323−339_ peptide as APCs and DO11.10 T cells stably expressing CCR7-YFP. Again, CCR7-YFP was readily recruited to the immunological synapse if BMDCs were loaded with both cognate antigen and one of the CCR7 ligands (Supplementary Figures 1E,F). In the absence of chemokines, CCR7 accumulation at the immunological synapse was less pronounced in conjugates between T cells and DCs compared to those formed between T and B cells.

It was reported previously that “trapping” of CCR5 and CXCR4 at the immunological synapse reduced the responsiveness of T cells to the cognate chemokines (14). To test whether this also holds true for CCR7, we performed Transwell migration assays with 3B11 T cells expressing CCR7-YFP incubated or not with HEL antigen pulsed or unpulsed LK35.2 APCs. 3B11 T cells or 3B11 T cells incubated with unpulsed LK35.2 APCs migrated similarly toward CCL19 or CCL21 (Supplementary Figure 1D). In contrast, 3B11 T cells incubated with HEL antigen pulsed LK35.2 APCs barely migrated toward CCR7 ligands (Supplementary Figure 1D), suggesting that T cells conjugated to cognate antigen-bearing APCs show reduced chemotactic responses toward CCR7 ligands.

In summary, we observed an unexpected context specific redistribution and enrichment of CCR7, particularly in plasma membrane adjacent vesicles, at the immunological synapse under TCR and CCR7 co-stimulatory conditions.

### CCR7 Interacts With the TCR CD3ζ Chain

To characterize the co-stimulatory function of the chemokine receptor we investigated whether CCR7 co-localized with CD3ζ of the TCR complex. We observed a partial co-localization of CCR7-YFP with CD3ζ in 3B11 T cells that significantly increased upon CCR7 stimulation (Figure 1E, Supplementary Figure 2A), similarly to what was reported for CXCR4 and CD3ζ (29). Co-localization between CCR7-YFP and CD3ζ further increased in 3B11 T cells if conjugated to HEL-loaded LK35.2 APCs and was highest if in addition CCR7 was triggered by its ligands (Figure 1E, Supplementary Figure 2A). Again, CCR7-YFP was readily found at the plasma membrane and at vesicular structures after chemokine addition (Figure 1E, Supplementary Figure 2A). To confirm the interaction of endogenous CCR7 and CD3, we performed proximity ligation assay (PLA) in primary human PBLs and found that the PLA signal significantly increased in PBLs upon stimulation with CCL19 or CCL21 (Supplementary Figure 2B). In addition, we analyzed the interaction between CCR7 and CD3ζ using YFP-bimolecular fluorescence complementation (BiFC) (17). To do so, CCR7 and CD3ζ were fused to non-fluorescent splitYFP1 (YFP1) and splitYFP2 (YFP2), respectively, and expressed in HEK293 cells. Upon interaction of CCR7 with CD3ζ the two splitYFP fragments reconstitute to form native YFP. BiFC between CCR7-YFP1 and CD3ζ-YFP2 was visualized by confocal microscopy (Supplementary Figure 2C) and quantified by flow cytometry (Figure 1F). In line with our co-localization and PLA data, we provide evidence that CCR7 directly interacts with CD3ζ of the TCR complex, but not with the GABA receptor Gat1, that served as a negative control (Figure 1F). Moreover, chemokine triggering substantially enhanced CCR7:CD3ζ BiFC (Figure 1F). Notably, the interaction between CCR7 and the TCR was not restricted to the plasma membrane, but was also observed in vesicular structures, particularly after chemokine stimulation.

### CCR7 Triggering Recruits and Activates ZAP70

**Figure 2:**
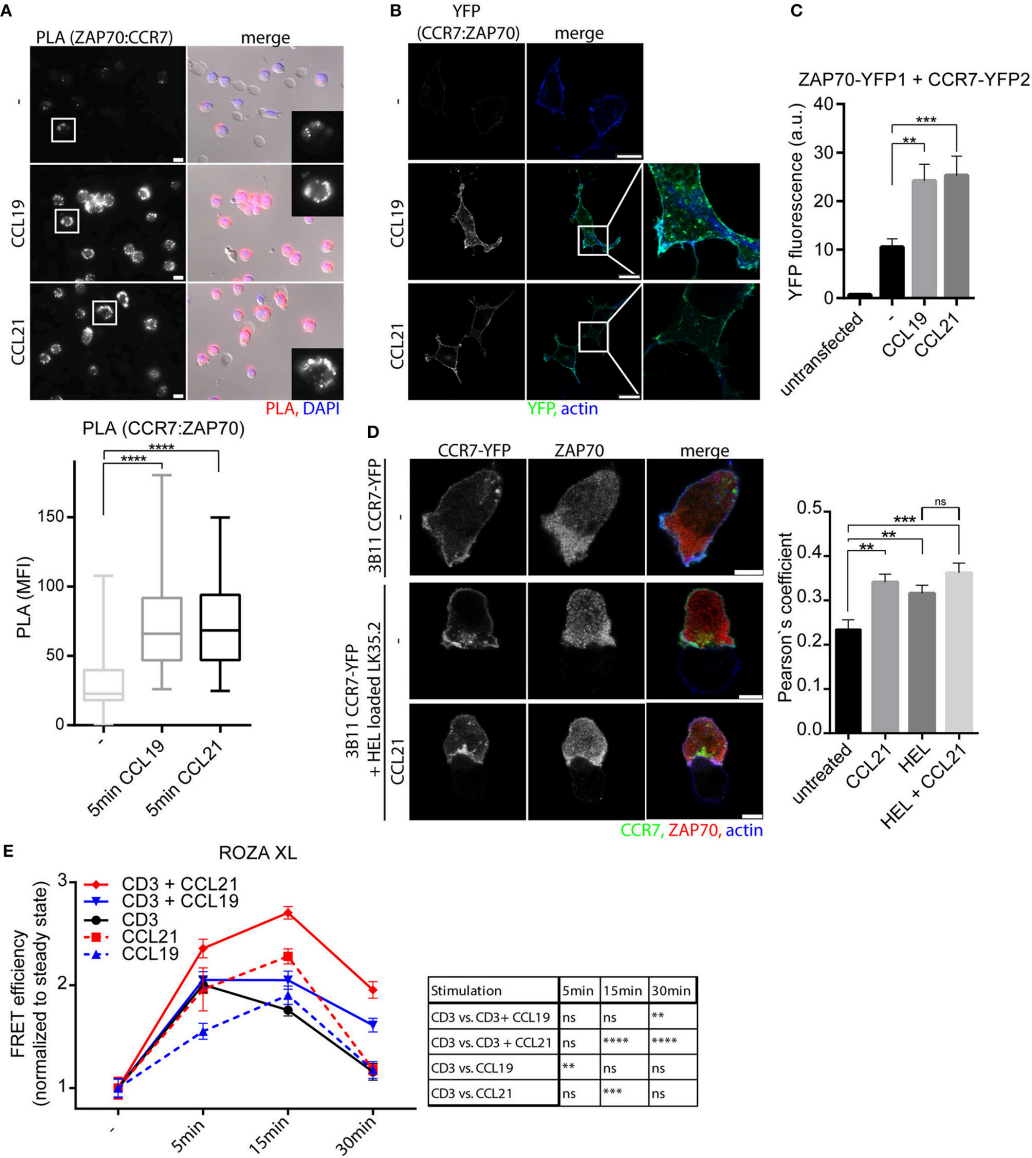
CCR7 triggering recruits and activates ZAP70. **(A)** Micrograph and quantification (box and whisker plot, *n* > 50 cells) of CCR7-ZAP70 interaction in human PBLs stimulated or not with 0.5 μg/ml CCL19 or CCL21 for 15 min assessed by PLA. Representative epifluorescence images derived from one donor out of three; scale bar, 10 μm. **(B)** Confocal images of CCR7-YFP2:ZAP70-YFP1 interaction determined by BiFC in transfected HEK293 cells stimulated or not with 0.5 μg/ml CCL19 or CCL21 for 1 h. One experiment out of three; scale bars, 25 μm. **(C)** Quantification of CCR7-YFP2:ZAP70-YFP1 interaction by BiFC assessed by flow cytometry after 1 h stimulation with 0.5 μg/ml CCL19 or CCL21. Mean ± SEM of five independent experiments. **(D)** Confocal images and quantification (Pearson's coefficient, *n* > 15 cells) of the co-localization between CCR7-YFP and ZAP70 in 3B11 T cells stimulated or not with 0.5 μg/ml CCL21 for 1 h either alone or conjugated with HEL peptide-loaded LK35.2 APCs. Representative images derived from one of three independent experiments; scale bar, 5 μm. **(E)** Quantification of ZAP70 activation determined by FRET efficiency of the ROZA-XL FRET biosensor in Jurkat T cells upon CCR7 and/or TCR stimulation [5 μg/ml anti-CD3 (clone OKT3)]. Mean ± SEM of 10 cells per condition derived from one out of three independent experiments. ^**^p < 0.01; ^***^p < 0.001; ^****^p < 0.0001. ns, not significant.

In a protein-protein-interaction screen, we recently identified that both SH2 domains of ZAP70 individually interacted with activated, tyrosine phosphorylated CCR7 (17). Whether the full ZAP70 protein, rather than just its SH2 domains, interacts with CCR7 and whether ZAP70 contributes to CCR7 signaling has not been investigated. Here, we used PLA to confirm that endogenous CCR7 and ZAP70 interact with each other in human PBLs and observed a significant chemokine-driven recruitment of ZAP70 to CCR7 (Figure 2A). A chemokine-driven recruitment of ZAP70 to CCR7 was also found by BiFC between ZAP70-YFP1 and CCR7-YFP2 in HEK293 cells (Figures 2B,C). Again, chemokine-mediated CCR7:ZAP70 BiFC was observed at the plasma membrane as well as at intracellular vesicular structures (Figure 2B). As HEK293 cells do not express TCR, these results suggest that TCR signaling is dispensable for the recruitment of ZAP70 to CCR7, but depends on chemokine stimulation. In 3B11 T cells, CCR7-YFP partially co-localized with ZAP70 and co-localization increased if T cells were conjugated with antigen-loaded APCs and/or stimulated with chemokines (Figure 2D, Supplementary Figures 3A,E). Notably, active ZAP70, phosphorylated at Y319, was predominantly found at the immunological synapse where it partially co-localized with CCR7 (Supplementary Figures 3B,C). To investigate whether CCR7 triggering results in ZAP70 activation, we used the FRET-based biosensor ROZA-XL, a reporter for ZAP70 tyrosine kinase activity (27) that we expressed in Jurkat T cells. Interestingly, CCR7 triggering in these Jurkat T cells resulted in increased ZAP70 kinase activity which was maximal after 15 min and subsequently returned to basal levels after 30 min of chemokine stimulation (Figure 2E). No increase in the FRET signal was measured in Jurkat T cells expressing the control sensor ROZA-XL YF (27), where the tyrosine residue in the substrate domain to be phosphorylated by ZAP70 is mutated (Supplementary Figure 3D). OKT3-mediated stimulation of the TCR revealed a different kinetic of ZAP70 kinase activity with a maximum FRET efficiency after 5 min of stimulation which returned to basal activity after 30 min (Figure 2E). Remarkably, CCR7 and TCR co-stimulation revealed a significantly increased ZAP70 kinase activity for CCL21 stimulation and a significantly prolonged ZAP70 tyrosine kinase activity for both, CCL19 and CCL21 stimulation (Figure 2E). In summary, we found that ZAP70 is recruited to CCR7 in a chemokine-dependent manner. Furthermore, we demonstrate that CCR7 and TCR co-stimulation results in enhanced and prolonged ZAP70 kinase activity, which likely contributes to the co-stimulatory function of CCR7.

### Chemokine-Driven ZAP70 Recruitment to CCR7 Depends on Src Kinase

**Figure 3:**
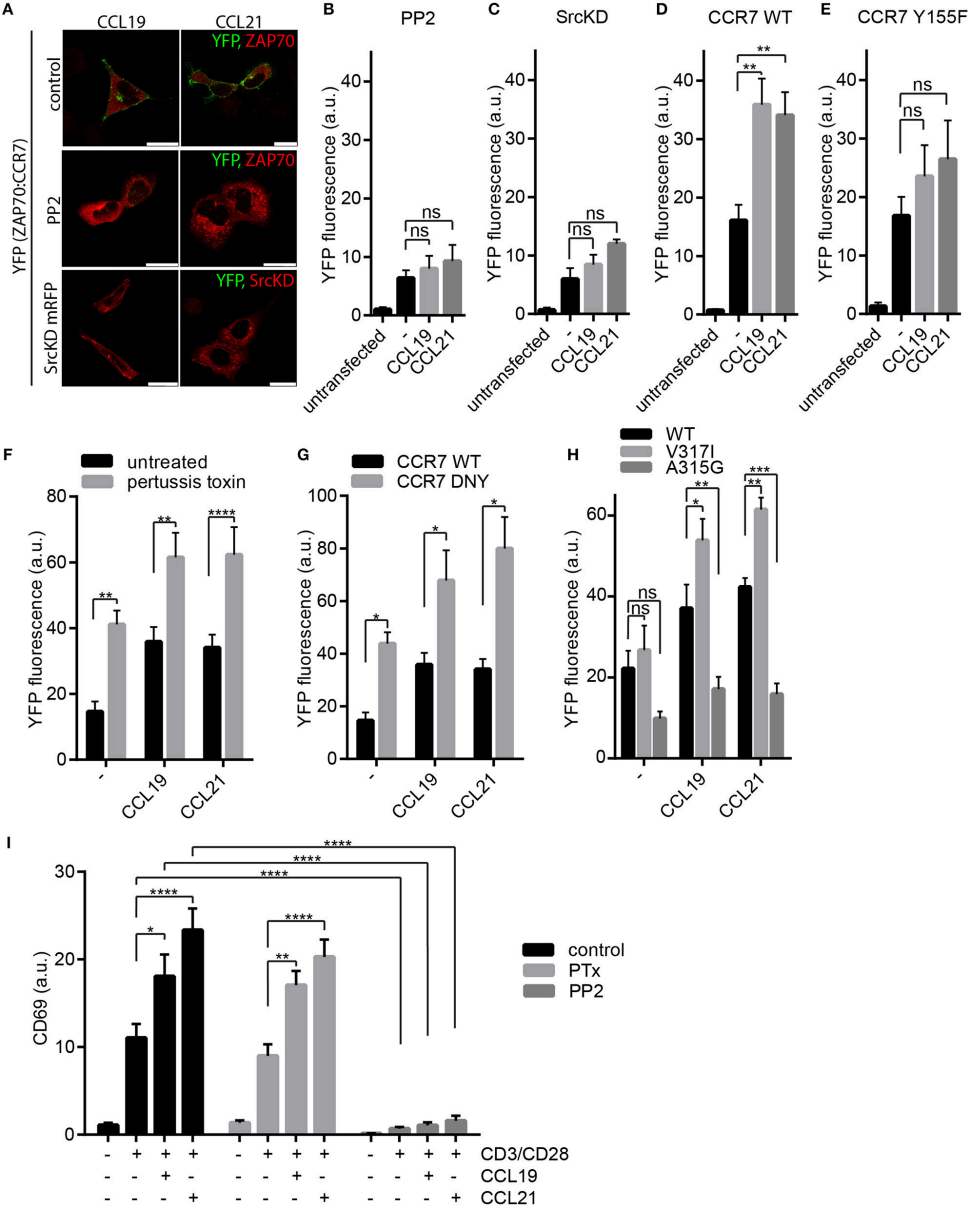
Chemokine-driven ZAP70 recruitment to CCR7 depends on Src. **(A)** Confocal images of CCR7-YFP2:ZAP70-YFP1 BiFC upon chemokine stimulation (0.5 μg/ml for 1 h) in HEK293 cells that were left untreated or pre-treated with PP2 (10 μM), or co-transfected with a kinase-dead variant of Src (SrcKD). Representative images derived from one out of three independent experiments; scale bars, 25 μm. **(B,C)** Quantification of CCR7-YFP2:ZAP70-YFP1 BiFC assessed by flow cytometry in transfected HEK293 cells pre-treated or not with PP2 (10 μM) **(B)** or co-transfected with SrcKD **(C)**. Mean ± SEM of three **(C)** or four **(B)** independent experiments. Cells were stimulated with 0.5 μg/ml CCL19 or CCL21 for 1 h. **(D,E)** Quantification of CCR7-YFP2:ZAP70-YFP1 **(D)** and CCR7-Y155F-YFP2:ZAP70-YFP1 **(E)** interaction determined by BiFC and flow cytometry in transfected HEK293 cells that were stimulated or not with 0.5 μg/ml CCL19 or CCL21 for 1 h. Mean ± SEM of three independent experiments. **(F)** Quantification of CCR7-YFP2:ZAP70-YFP1 BiFC in HEK293 cells treated or not with PTx (100 ng/ml) in presence or absence of 0.5 μg/ml CCL19 or CCL21 for 1 h. Mean ± SEM of three independent experiments. **(G)** Quantification of CCR7-DNY-YFP2:ZAP70-YFP1 assessed by BiFC and flow cytometry in transfected HEK293 cells. Mean ± SEM of three independent experiments. **(H)** Quantification of CCR7 WT, CCR7-V317I, or CCR7-A315G fused to YFP2 interaction with ZAP70-YFP1 determined by BiFC and flow cytometry in transfected HEK293 cells. Cells were stimulated or not with 0.5 μg/ml CCL19 or CCL21 for 1 h. Mean ± SEM of four independent experiments. **(I)** Quantification of CD69 surface expression on human PBLs, pretreated with PTx (100 ng/ml) or PP2 (10 μM) or left untreated, upon TCR stimulation using anti-CD3/CD28 coated beads and/or CCR7 stimulation (0.5 μg/ml CCL19 or CCL21) for 20 h measured by flow cytometry. Mean ± SEM of four individual donors. ^*^p < 0.1; ^**^p < 0.01; ^***^p < 0.001; ^****^p < 0.0001. ns, not significant.

Next, we aimed to decipher the signaling pathway regulating the recruitment of ZAP70 to CCR7. CCR7 signals initiated at the plasma membrane can be transmitted in a G protein-dependent and G protein-independent manner (17). Latter involves Src kinase (17) and can be mediated by β-arrestins (30) which can act as scaffold proteins besides facilitating receptor desensitization (31). To discriminate between the G protein-dependent and -independent signaling pathways, we exploited the BiFC system to monitor chemokine-driven ZAP70-YFP1 recruitment to CCR7-YFP2 in HEK293 cells (Figures 2B,C, Figure 3A). Pharmacological inhibition of Src kinase activity using PP2 abolished chemokine-driven ZAP70 recruitment to CCR7 (Figures 3A,B). Similarly, overexpressing a kinase-dead (KD) mutant form of Src also prevented chemokine-driven CCR7:ZAP70 BiFC (Figures 3A,C). Moreover, mutating Y155 of CCR7 known to be phosphorylated by Src (17) reduced chemokine-triggered CCR7 ZAP70 interaction (Figures 3D,E, Supplementary Figures 4A,B). Notably, chemokine-driven CCR7:ZAP70 BiFC was observed in mouse embryonic fibroblasts (MEFs) derived from wild-type as well as β-arrestin 1 and 2 double deficient mice (Supplementary Figure 4C), demonstrating that β-arrestins are dispensable for chemokine-mediated recruitment of ZAP70 to CCR7. To interfere with the G protein-dependent CCR7 signaling pathway, we pharmacologically blocked G_i_ protein-coupling using pertussis toxin (PTx) and additionally exploited the CCR7-DNY mutant that is unable to couple to G proteins (25). To our surprise, both pharmacological as well as genetic interference with G protein-coupling resulted in a significantly higher BiFC signal between CCR7-YFP2 and ZAP70-YFP1 in cells not exposed to chemokines (Figures 3F,G). Stimulation of these cells with CCL19 or CCL21 further increased CCR7:ZAP70 BiFC (Figures 3F,G). We have shown recently that Src constantly interacts with oligomeric CCR7, where the kinase is autophosphorylated upon CCR7 engagement, and that oligomeric CCR7 is able to integrate G protein and Src-dependent signaling (17). Hence, we speculated that pharmacological and genetic uncoupling of the G protein from the receptor might render CCR7 more accessible for Src and thereby facilitate CCR7 ZAP70 interaction. To test this hypothesis, we made use of CCR7 point mutants owing either oligomerization-defective (CCR7 A315G) or super-oligomerization (CCR7 V317I) characteristics, manifested by reduced or enhanced chemokine-driven Src activity, respectively (17). In our BiFC assay, we found that chemokine-triggered recruitment of ZAP70 was significantly higher in cells expressing CCR7 V317I and significantly lower in cells expressed CCR7 A315G (Figure 3H). These data support our hypothesis that interfering with G protein-coupling renders CCR7 more accessible to interact with Src and, consequently with ZAP70. In addition, these data provide evidence that chemokine-induced ZAP70 recruitment to CCR7 involves Src kinase activity.

To address the role of Gα_i_ signaling in CCR7-driven T cell co-stimulation, we pre-treated primary human PBLs with PTx prior to CD3/CD28 triggering in the presence or absence of chemokines. We found that PTx treatment did not affect CCR7-driven T cell co-stimulation as determined by CD69 surface expression (Figure 3I). In contrast, treating PBLs with PP2 abolished CD69 induction independent of CCR7 co-stimulation (Figure 3I), which is in line with the essential role of Src kinases in TCR signaling and T cell activation (32).

### CCR7 Stimulation Promotes the Interaction of ZAP70 With Vav1

**Figure 4:**
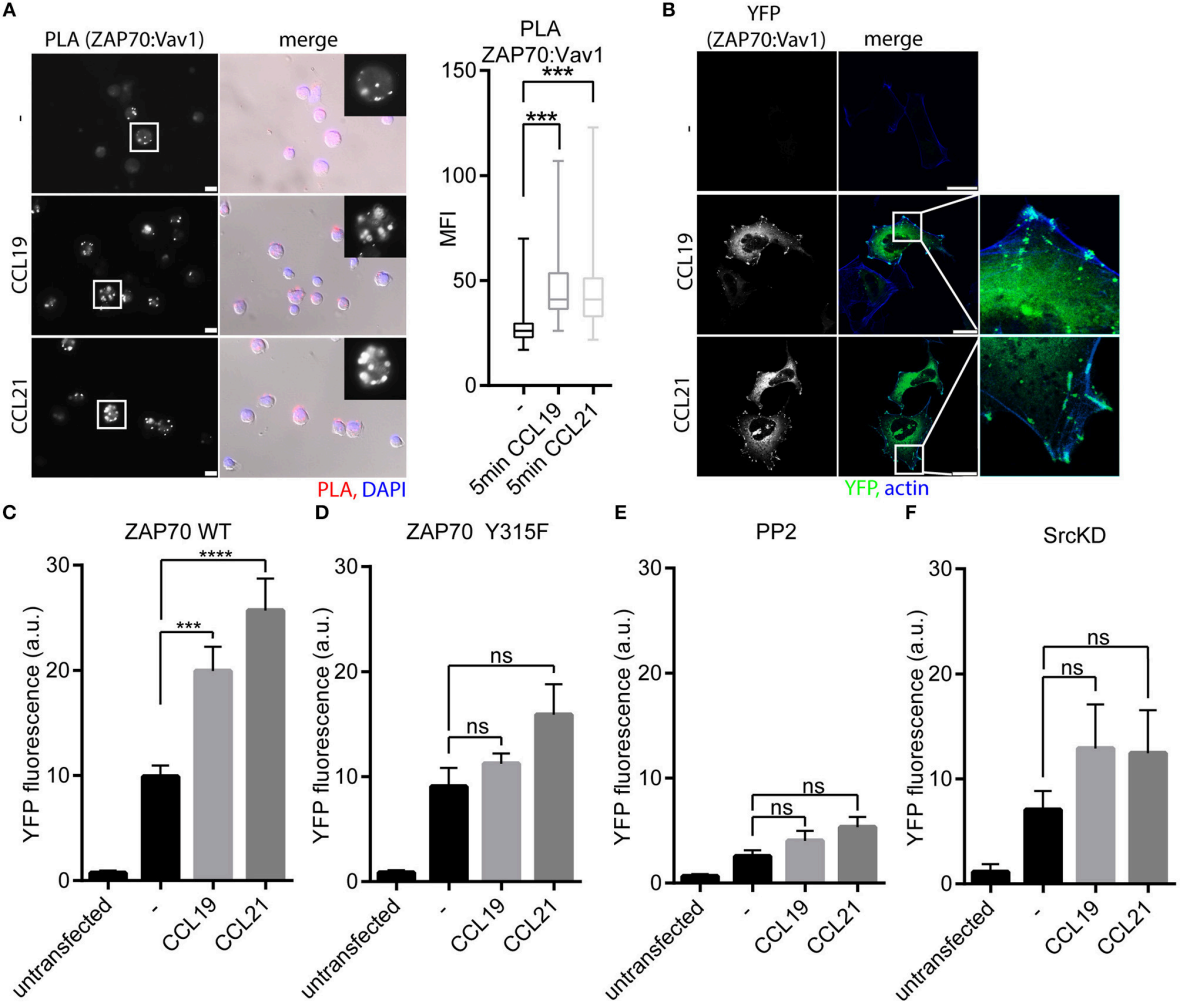
Vav1 recruitment to ZAP70 upon CCR7 stimulation depends on Src kinase and Y315-phosphorylated ZAP70. **(A)** Micrograph and quantification (box and whisker plot, *n* > 35 cells) of Vav1-ZAP70 interaction in primary human PBLs assessed by PLA. Cells were stimulated or not with 0.5 μg/ml CCL19/CCL21 for 15 min. Representative epifluorescence images derived from one donor out of three; scale bar, 10 μm. **(B)** Confocal images of ZAP70-YFP1:Vav1-YFP2 interaction determined by BiFC in HEK293 cells expressing CCR7-HA upon stimulation with CCL19 or CCL21 (0.5 μg/ml, 1 h). Representative images derived from one out of three independent experiments; scale bar, 25 μm **(C)** Quantification of ZAP70-YFP1:Vav1-YFP2 interaction determined by BiFC and flow cytometry in HEK293 cells expressing CCR7-HA stimulated with CCL19 or CCL21 (0.5 μg/ml, 1 h). Mean ± SEM of five independent experiments. **(D)** Quantification of ZAP70-Y315F-YFP1:Vav1-YFP2 interaction determined by BiFC and flow cytometry in HEK293 cells expressing CCR7-HA stimulated with CCL19 or CCL21 (0.5 μg/ml, 1 h). Mean ± SEM of three independent experiments. **(E,F)** Quantification of ZAP70-YFP1:Vav1-YFP2 interaction assessed by BiFC and flow cytometry in HEK293 cells expressing CCR7-HA after PP2 (10 μM) treatment **(E)** or SrcKD co-transfection **(F)**. Mean ± SEM of three **(F)** or four **(E)** independent experiments. ^***^p < 0.001; ^****^p < 0.0001. ns, not significant.

Inspired by the fact that ZAP70 is recruited to CCR7, and that ZAP70 tyrosine kinase is activated downstream of CCR7, we further characterized the CCR7 signaling pathway involving ZAP70. Therefore, we next addressed the role of the Rac/RhoGEF Vav1, a known interaction partner of ZAP70 downstream of the TCR (33) and CXCR4 (19). Indeed, CCR7 triggering in human PBLs significantly increased the interaction of ZAP70 with Vav1 as determined by PLA (Figure 4A). Recruitment of Vav1 to ZAP70 upon chemokine stimulation was affirmed by BiFC in HEK293 cells expressing ZAP70-YFP1 and Vav1-YFP2 together with CCR7-HA (Figure 4B). Quantification of ZAP70:Vav1 BiFC by flow cytometry revealed that CCR7 triggering significantly enhanced the interaction between ZAP70 and Vav1 (Figure 4C). As downstream of the TCR, ZAP70 Vav1 interaction depends on Y315-phosphorylation of ZAP70 (18), we generated the ZAP70 Y315F mutant fused to YFP1. CCR7 triggering did not promote BiFC between ZAP70 Y315F-YFP1 with Vav1-YFP2 as quantified by flow cytometry (Figure 4D), indicating that CCR7-driven Vav1 recruitment to ZAP70 depends on Y315-phosphorylation of the kinase. In line with this, chemokine-mediated ZAP70:Vav1 BiFC was abolished by PP2 treatment (Figure 4E) or by overexpressing SrcKD (Figure 4F). These data suggest that CCR7 triggering primarily activates Src, which is required for ZAP70 phosphorylation and the subsequent recruitment of Vav1.

### ZAP70 Is Vital for CCR7-Driven LFA-1 Valency Regulation and T Cell Arrest on ICAM-1

**Figure 5:**
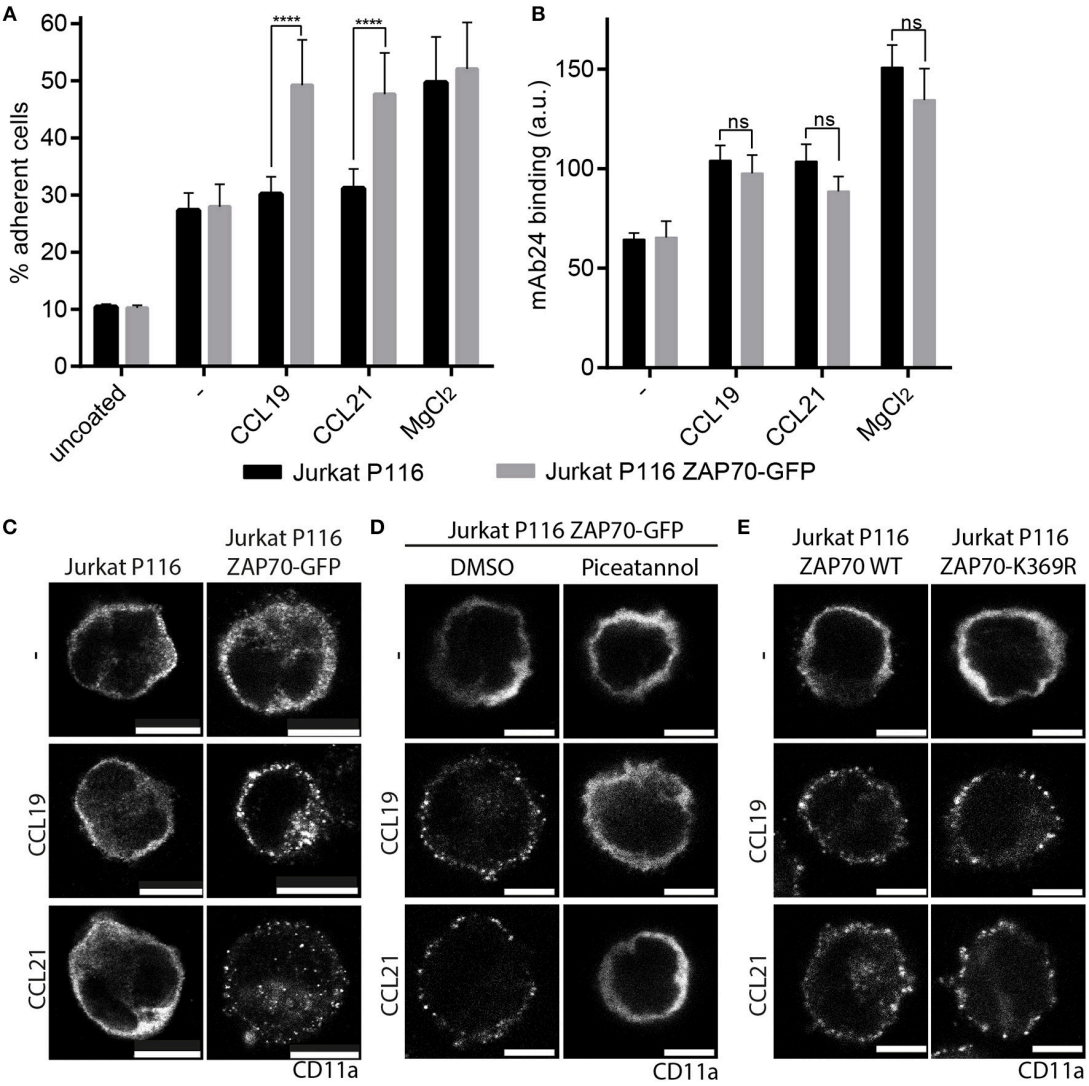
ZAP70 controls CCR7-mediated inside-out signaling to LFA-1. **(A)** Arrest of Jurkat P116 cells lacking ZAP70 and Jurkat P116 cells reconstituted with ZAP70-GFP on immobilized ICAM-1 in the presence or absence of CCL19, CCL21, or MgCl_2_. Mean ± SEM of three independent experiments. **(B)** Binding of the β_2_-integrin high-affinity reporter mAb24 to Jurkat P116 and Jurkat P116 ZAP70-GFP cells upon CCR7 triggering for 10 min. Mean ± SEM of five independent experiments. **(C–E)** Confocal images of CD11a staining on Jurkat P116, Jurkat P116 ZAP70-GFP, or Jurkat P116 ZAP70-K369R-GFP cells before and after chemokine stimulation (0.5 μg/ml, 10 min). **(C)** no pre-treatment. **(D)** pre-treatment with DMSO or piceatannol. Representative images derived from one out of three independent experiments; scale bar, 10 μm. ^****^p < 0.0001. ns, not significant.

ZAP70 is known to associate with LFA-1 and its inhibition was found to interfere with outside-in signaling and LFA-1-dependent T cell motility (34, 35). Hence, we addressed whether ZAP70 plays a role in CCR7-mediated inside-out signaling to integrins and subsequent T cell adhesion. To investigate this, we used Jurkat P116 T cells deficient in ZAP70-expression and control cells that were reconstituted with ZAP70-GFP (Supplementary Figures 5A–D). Jurkat P116 T cells devoid of ZAP70 were unable to transmit CCR7-mediated inside-out signals to LFA-1 as manifested by the lack of chemokine-driven cell adhesion to ICAM-1 (Figure 5A). In contrast, Jurkat P116 T cells reconstituted with ZAP70-GFP readily adhered to ICAM-1 upon CCR7 triggering (Figure 5A). Chemokine-driven inside-out signaling to integrins resulting in cell adhesion can be achieved by two ways, either by triggering a high-affinity conformation of the integrin or by integrin clustering (8). To analyze the effect of ZAP70 on LFA-1 affinity regulation, we used the β_2_-integrin specific high-affinity reporter antibody mAb24 (36). mAb24 binding increased significantly upon CCR7 triggering in Jurkat T cells regardless whether they expressed ZAP70 or not (Figure 5B). In contrast, CCR7-driven clustering of CD11a, the α-chain of LFA-1, was exclusively observed in Jurkat T cells expressing ZAP70-GFP, and no chemokine-induced CD11a clustering was observed in ZAP70 deficient Jurkat P116 cells (Figure 5C). In line with this, treatment of Jurkat P116 cells expressing ZAP70-GFP with piceatannol, which inhibits members of the Syk family, abrogated CCR7-induced CD11a clustering (Figure 5D). However, CCR7-triggered CD11a clustering was observed in Jurkat P116 T cells expressing a kinase-dead mutant of ZAP70 (ZAP70 K369R) (Figure 5E), indicating that the adaptor function of ZAP70, rather than its kinase activity confers CCR7-mediated LFA-1 clustering and T cell adhesion.

In summary, we found that CCR7 is recruited to the immunological synapse and acts as co-stimulatory molecule in human T cells manifested by enhanced IL-2 secretion and CD69 induction. We demonstrated that CCR7 triggering recruits and activates ZAP70. In addition, we provide experimental evidence that ZAP70 controls CCR7-driven inside-out signaling to LFA-1 by valency regulation of the integrin to promote T cell adhesion, a critical step in the formation of the immunological synapse.

## Discussion

The formation of a stable immunological synapse is key for efficient priming of T cells. The process of synapse formation and T cell activation is influenced by a range of factors and it consequently affects T cell proliferation and differentiation subsequent to T cell priming. Proper T cell activation necessitates not only signals by the TCR, but also from co-stimulatory molecules (37). Interestingly, the inflammatory chemokines CCL2, CCL3, and CCL5 are able to provide co-stimulatory signals to T cells and to influence T cell differentiation (8, 38–40). In addition, also the homoeostatic chemokine CXCL12 can act co-stimulatory on human T cells (29, 41, 42). Consequently, the receptors for these chemokines, namely CCR5 and CXCR4, are recruited to the immunological synapse to fulfill their co-stimulatory functions (14). Strikingly, co-stimulatory activities have also been attributed to the homeostatic chemokines CCL19 and CCL21, at least in the murine system (9, 43, 44) despite that their receptor CCR7 was reported to be excluded from the immunological synapse (14). In the present study, we demonstrate that CCR7 acts as a potent co-stimulatory molecule on human T cells as manifested by increased CD69 expression and IL-2 production. Consistent with its co-stimulatory role, we found that CCR7 is recruited to the immunological synapse. Notably, CCR7 recruitment to the immunological synapse requires antigen recognition by the TCR. In the present study, we used CCR7 fused intracellularly to YFP to monitor its recruitment to the immunological synapse, whereas Molon and colleagues used CCL21 fused to the Fc-portion of an antibody in combination with a fluorescently labeled secondary antibody to visualize CCR7 (14). The caveat of this indirect detection method may be that the reagents have only limited access to the tight interspace at the immunological synapse between the APC and T cell, whereas CCR7 outside the synapse is easily accessible. Using the same visualization strategy we used, Molon and colleagues demonstrated that CCR5-GFP and CXCR4-GFP are recruited to the immunological synapse upon antigen recognition (14). Nonetheless, Molon and co-workers found that chemokine receptor engagement enhanced T cell-APC adhesion, and T cells that were engaged with antigen-bearing APC were less responsive to chemokines and migrated less efficiently (14). This finding is in line with the “stop and go” hypothesis, proposing that stability and duration of the immunological synapse is determined by migratory “go” signals derived from chemokine receptors, and “stop” signals provided by the TCR (45). Consequently, T cells must be able to integrate and adapt to these opposing signals. In the present study, we observed that CCR7 is recruited to the immunological synapse like CCR5 and CXCR4. Notably, we found that chemokine triggering resulted in the accumulation of CCR7 in vesicles beneath the immunological synapse. Interestingly, there is growing evidence that sub-synaptic vesicles control signaling events at the immunological synapse by two ways: by concentrating and supplying signaling molecules in microclusters beneath the synapse and by acting as signaling platforms themselves (46). In line with this, we discovered that upon chemokine stimulation, CCR7 predominantly associates with the TCR and ZAP70 at sub-synaptic vesicles. So far, the only chemokine receptor that was described to interact with the TCR and to signal via ZAP70 is CXCR4 (29, 47, 48). Interestingly, CXCR4:TCR heterodimers are mainly observed in intracellular vesicles (29). However, chemokine stimulation of CXCR4 resulted in the recruitment of ZAP70 to ITAM motives of the TCR complex (29), whereas here we provide evidence that ZAP70 is recruited directly to phosphorylated Y155 of CCR7. Notably, we found that chemokine-driven ZAP70 recruitment to CCR7 also occurred in cells that do not express the TCR. That CCR7 directly interacts with ZAP70 is supported by the fact that both SH2 domains of ZAP70 were found in a screen for proteins containing SH2-domains that directly interacted with tyrosine-phosphorylated CCR7 (17). We show that the recruitment of ZAP70 to CCR7 depends on Src, which phosphorylates the chemokine receptor at Y155, but does not rely on G protein-coupling. This is in line with the co-stimulatory function of CCR5, which is also insensitive to PTx (14) and with the finding that CCL21-mediated T cell co-stimulation was not affected in mice lacking PI3Kγ or expressing a kinase-dead variant of PI3Kδ (9), as PI3Ks are activated in a G protein-dependent manner down-stream of chemokine receptors (49). Notably, T cell co-stimulation is reduced in mice lacking DOCK2, a RhoGEF down-stream of both the TCR and chemokine receptors (9). As signaling to DOCK2 by chemokine receptors is mediated by Gα_i_-coupling (50), it is unlikely that chemokine receptor signaling exploits DOCK2 directly to drive co-stimulation. Maybe DOCK2 acts on the cytoskeleton via Rac1 to influence synapse formation to modulate T cell co-stimulation. Along the CCR7 signaling pathway, we found that another RhoGEF, namely Vav1, is recruited to ZAP70 in a G protein-independent, but Src-dependent manner. It is interesting to note that CXCR4-induced ZAP70 activation results in Vav1 phosphorylation and its subsequent dissociation from talin. This allows talin to interact with VLA-4 such that the integrin can attain the high-affinity conformation required for T cell adhesion (19–21). Here, we show that CCR7 triggering leads to the recruitment of Vav1 to ZAP70 through Src. Acting as adaptor protein we found ZAP70 to be essential for CCR7-mediated inside-out signaling to LFA-1 resulting in integrin clustering. Consequently, T cells lacking ZAP70 failed to cluster LFA-1 in response to CCR7 triggering and adhered less to ICAM-1. Thus, it is conceivable that CCR7, utilizing ZAP70 as adapter protein, contributes to the stabilization of the immunological synapse by inducing clustering of the integrin LFA-1. This scenario is supported by the fact that LFA-1 clustering is not only important for adhesion, but also for the formation of intervening LFA-1 free areas in which the TCR scans the surface of APCs for cognate antigens (51), supporting T cell activation. In addition to its function as adaptor protein in integrin inside-out signaling, we provide evidence that ZAP70 kinase is activated upon CCR7 triggering. Importantly, we found enhanced and prolonged ZAP70 kinase activity under TCR CCR7 co-stimulation. As ZAP70 is one of the most up-stream elements in TCR signaling enhancement of ZAP70 kinase activity may contribute to the co-stimulatory function of CCR7.

## Author Contributions

DL and JL designed research and wrote the manuscript. JL, MA, and IK performed all experiments and analyzed the data. AP was responsible for collecting blood. DL supervised the overall study.

### Conflict of Interest Statement

The authors declare that the research was conducted in the absence of any commercial or financial relationships that could be construed as a potential conflict of interest.
