# Supplementary material for: CCR7 Is Recruited to the Immunological Synapse, Acts as Co-stimulatory Molecule and Drives LFA-1 Clustering for Efficient T Cell Adhesion Through ZAP70
Source: Front Immunol. 2019 Jan 14;9:3115. doi: 10.3389/fimmu.2018.03115 (PMC6339918; doi:10.3389/fimmu.2018.03115)
Supplement: Supplementary file 1 [file Data_Sheet_1.PDF]

Supplementary Material

CCR7 is recruited to the immunological synapse, acts as co-stimulatory molecule and drives LFA-1 clustering for efficient T cell adhesion through ZAP70

Julia M. Laufer, Ilona Kindinger, Marc Artinger, Andreas Pauli, and Daniel F. Legler*

*** Correspondence:** Daniel F. Legler: daniel.legler@bitg.ch

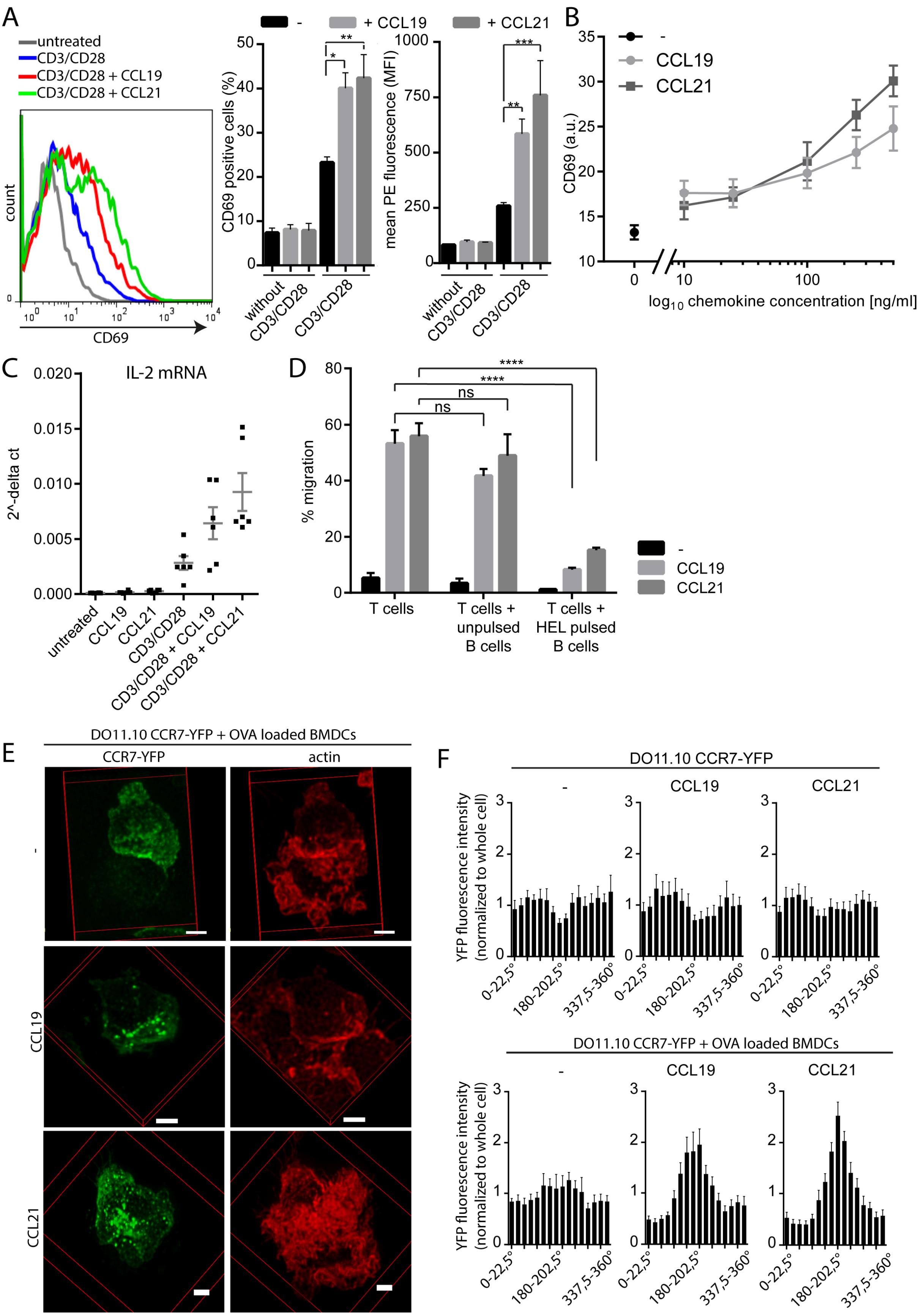
A
untreated
CD3/CD28
CD3/CD28 + CCL19
CD3/CD28 + CCL21
count
CD69
- + CCL19 + CCL21
CD69 positive cells (%)
mean PE fluorescence (MFI)
without CD3/CD28
CD3/CD28
B
CCL19
CCL21
CD69 (a.u.)
log10 chemokine concentration [ng/ml]
C
IL-2 mRNA
2^-delta ct
untreated
CCL19
CCL21
CD3/CD28
CD3/CD28 + CCL19
CD3/CD28 + CCL21
D
% migration
ns
T cells
T cells + unpulsed B cells
T cells + HEL pulsed B cells
E
DO11.10 CCR7-YFP + OVA loaded BMDCs
CCR7-YFP
actin
F
DO11.10 CCR7-YFP
YFP fluorescence intensity (normalized to whole cell)
0-22,5°
180-202,5°
337,5-360°
DO11.10 CCR7-YFP + OVA loaded BMDCs

Supplementary Figure 1. Co-stimulatory function of CCR7 and localization of CCR7 at the immunological synapse. (A) Quantification of CD69 surface expression by flow cytometry on primary human PBLs upon TCR stimulation using anti-CD3/CD28 coated beads and/or CCR7 stimulation by 0.5µg/ml CCL19 or CCL21 for 20h. Representative histogram (left) derived from one out of four experiments. Percent of CD69 positive human PBLs (middle) or mean CD69-PE fluorescence (right) of human PBLs 20h post stimulation. Mean ± SEM derived from four independent donors measured in triplicates. (B) Quantification of CD69 surface expression by flow cytometry on PBLs upon stimulation with anti-CD3/CD28 coated beads and CCR7 co-stimulation with graded concentrations of CCL19 or CCL21 (0.01µg/ml, 0.025µg/ml, 0.1µg/ml, 0.250µg/ml, 0.5µg/ml). Mean ± SEM derived from six independent donors. (C) Relative IL-2 mRNA expression in primary human PBLs after TCR and/or CCR7 triggering for 24h determined by quantitative real-time PCR was calculated using the 2^-delta Ct method. Mean and individual values derived from six individual donors. (D) Transwell migration assay of either 3B11 CCR7-YFP T cells alone or 3B11 CCR7-YFP T cells pre-incubated with LK35.2 APCs that were pulsed with HEL antigen or left unpulsed as control. Mean ± SEM of three independent experiments. (E) Deconvoluted 3D reconstructions of CCR7-YFP in DO11.10 T cells conjugated to OVA peptide loaded BMDCs for 1h in presence or absence of 0.5µg/ml of chemokines. Scale bar, 3µm. (F) Distribution and quantification of CCR7-YFP in DO11.10 T cells conjugated or not to OVA peptide loaded BMDCs stimulated or not with chemokines in Rose plot sectors. Analysis of 15 cells derived from three independent experiments; scale bar, 3µm.

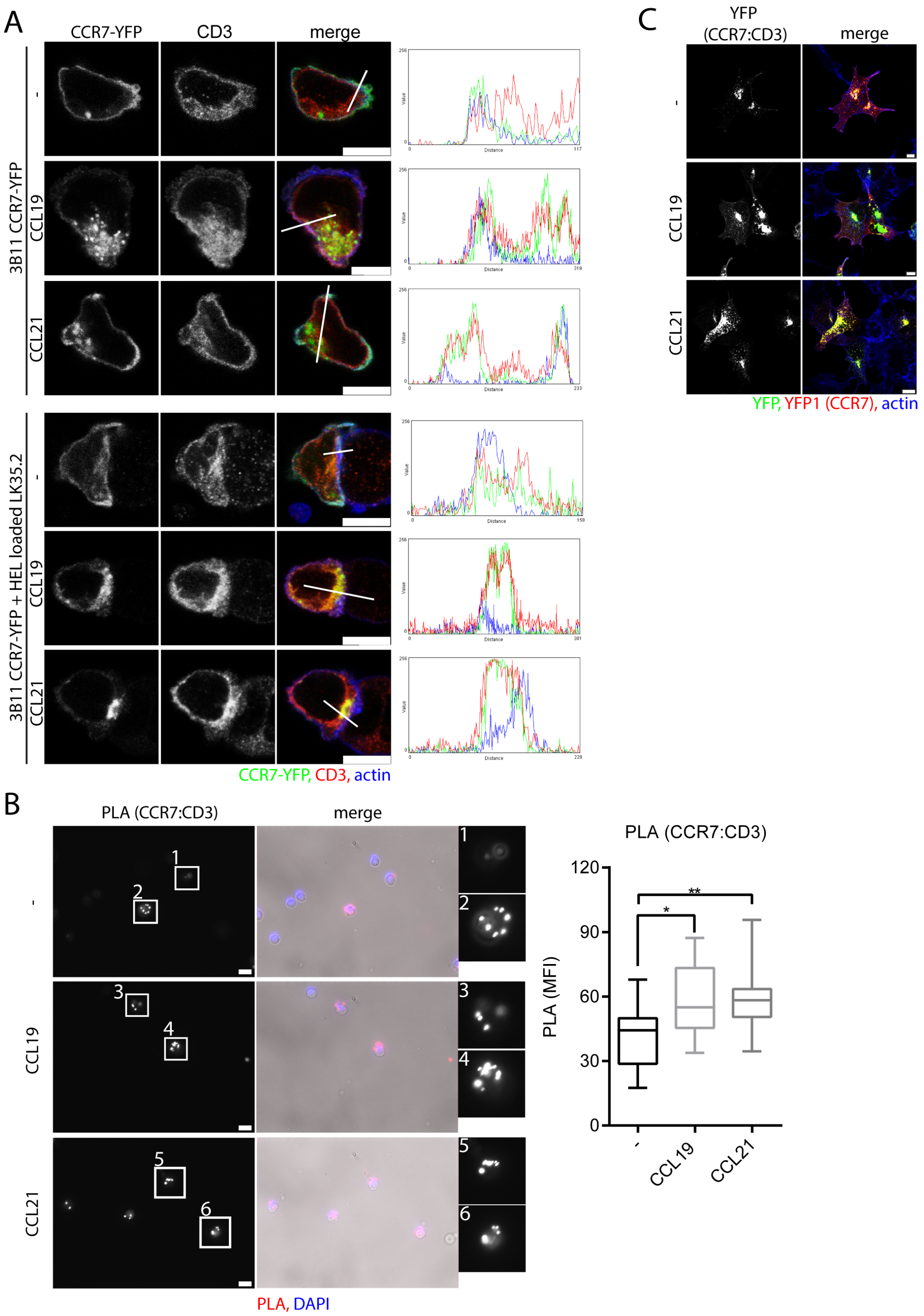

A
CCR7-YFP
CD3
merge
3B11 CCR7-YFP
-
CCL19
CCL21
3B11 CCR7-YFP + HEL loaded LK35.2
CCR7-YFP, CD3, actin
B
PLA (CCR7:CD3)
merge
PLA, DAPI
PLA (CCR7:CD3)
PLA (MFI)
120
90
60
30
0
*
**
CCL19
CCL21
C
YFP (CCR7:CD3)
YFP, YFP1 (CCR7), actin

Supplementary Figure 2. CCR7 associate with the CD3ζ chain of the TCR complex. (A) Line plot analysis of co-localization between CCR7-YFP and CD3ζ in 3B11 T cells upon 1h stimulation with chemokines (0.5µg/ml) alone or conjugated or not to HEL peptide-loaded LK35.2 APCs. One representative cell derived from one out of three independent experiments; scale bars, 7.5µm. (B) Micrograph and quantification (box and whisker plot, n > 50 cells) of endogenous CCR7-CD3ζ interaction in human PBLs stimulated or not with 0.5µg/ml CCL19 or CCL21 for 15min assessed by PLA. One experiment out of three; scale bars, 10µm. *$p<0.05$; **$p<0.01$; ***$p<0.001$. (C) Confocal images of CCR7-CD3ζ interaction determined by BiFC in transfected HEK293 cells upon stimulation with 0.5µg/ml CCL19 or CCL21 for 30min. One experiment out of three; scale bars, 10µm.

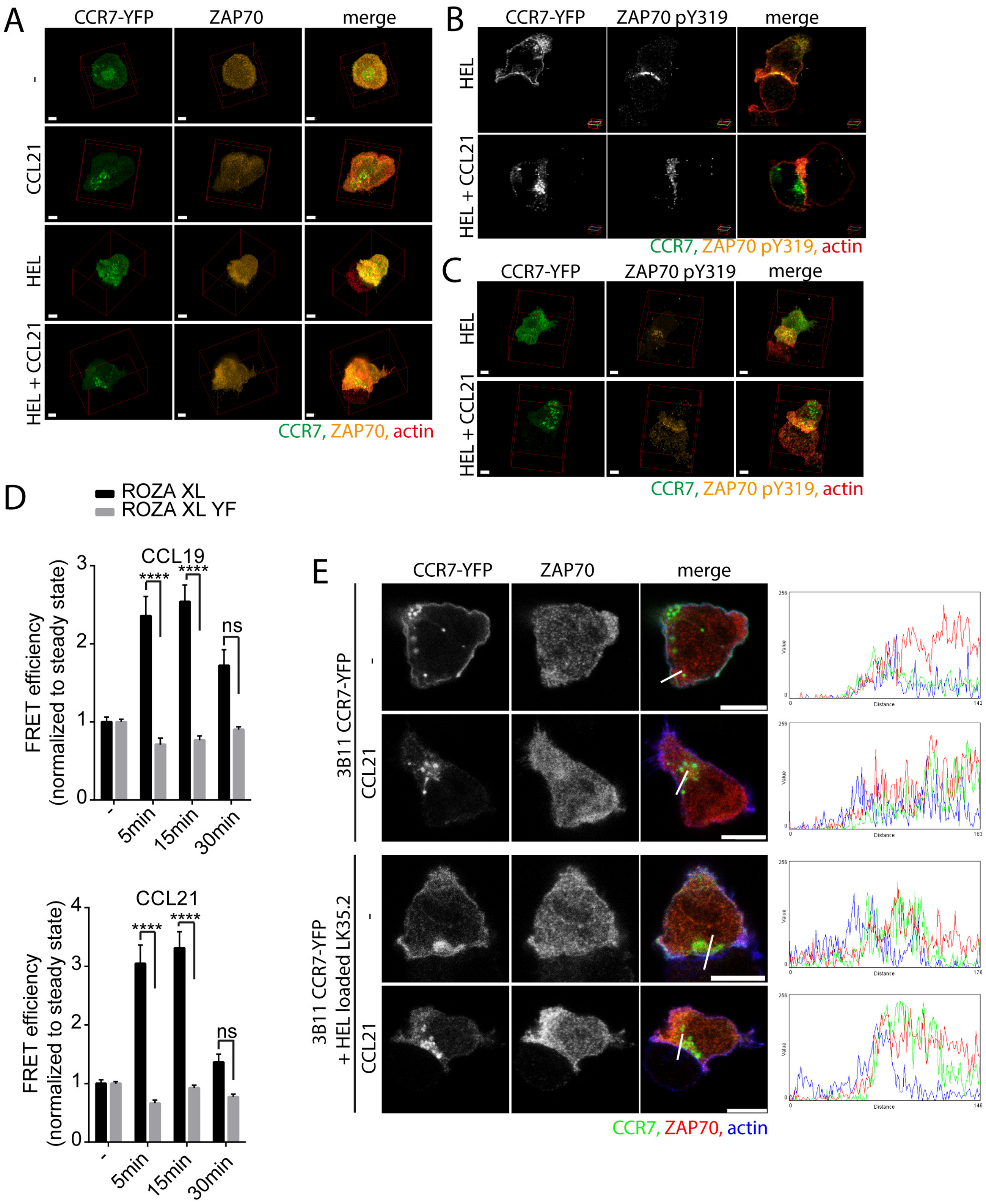
A
CCR7-YFP
ZAP70
merge
-
CCL21
HEL
HEL + CCL21
CCR7, ZAP70, actin
B
CCR7-YFP
ZAP70 pY319
merge
HEL
HEL + CCL21
CCR7, ZAP70 pY319, actin
C
CCR7-YFP
ZAP70 pY319
merge
HEL
HEL + CCL21
CCR7, ZAP70 pY319, actin
D
ROZA XL
ROZA XL YF
CCL19
FRET efficiency
(normalized to steady state)
****
****
ns
-
5min
15min
30min
CCL21
FRET efficiency
(normalized to steady state)
****
****
ns
-
5min
15min
30min
E
CCR7-YFP
ZAP70
merge
3B11 CCR7-YFP
-
CCL21
3B11 CCR7-YFP
+ HEL loaded LK35.2
-
CCL21
CCR7, ZAP70, actin

Supplementary Figure 3. Localization and activation of ZAP70 upon CCR7 triggering. (A) 3D reconstruction of CCR7-YFP and ZAP70 localization in 3B11 T cells after 1h incubation in the presence or absence of HEL peptide-loaded LK35.2 APCs and/or 0.5µg/ml CCL21. Scale bar, 3µm. (B) Deconvoluted single z-stack and (C) 3D reconstruction of confocal images of CCR7-YFP and Y319-phosphorylated ZAP70 in 3B11 T cells analyzed after 1h incubation in the presence or absence of HEL peptide-loaded LK35.2 APCs and/or 0.5µg/ml CCL21. Representative images derived from one out of three experiments; scale bar, 3µm. (D) Quantification of the ZAP70 kinase activity upon TCR stimulation (5µg/ml anti-CD3 (clone OKT3)) and/or chemokine stimulation determined by FRET efficiency of the ROZA-XL biosensor and its control sensor ROZA-XL-YF in transfected Jurkat cells. Mean ± SEM of 10 cells per condition derived from one out of three independent experiments. ****$p<0.0001$, ns not significant. (E) Line plot analysis of co-localization between CCR7-YFP and ZAP70 in 3B11 T cells upon incubation for 1h with 0.5µg/ml CCL21 alone and/or upon conjugation to HEL peptide-loaded LK35.2 APCs. One representative cell from one out of three independent experiments; scale bars, 7.5µm.

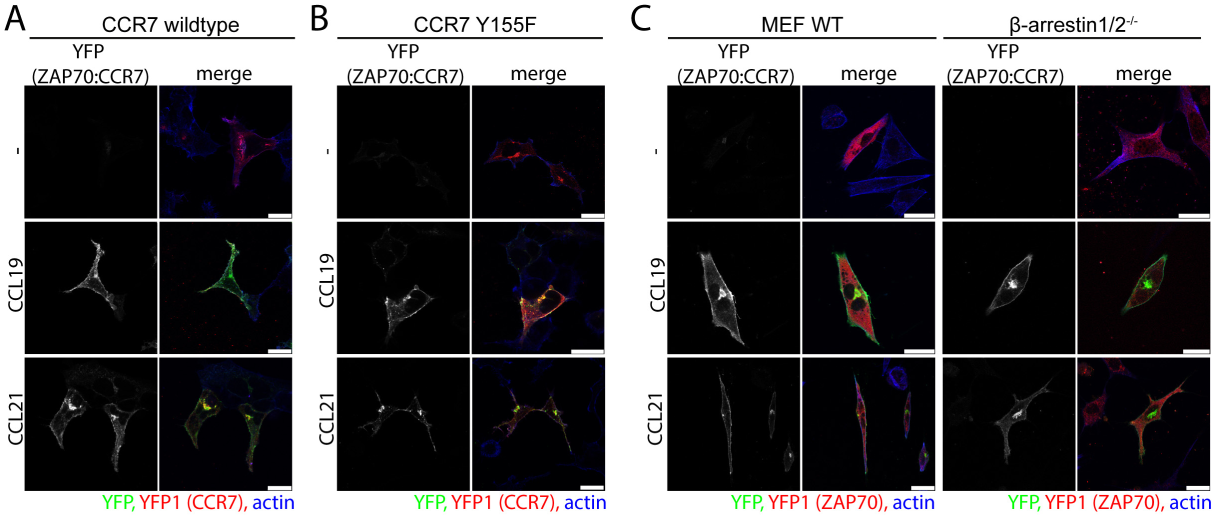

Supplementary Figure 4. Chemokine-driven ZAP70 recruitment to CCR7 depends on tyrosine 155 of CCR7 but is independent of β-arrestins. (A/B) Confocal images of CCR7-YFP1:ZAP70-YFP2 BiFC (A) or CCR7-Y155F-YFP1:ZAP70-YFP2 BiFC (B) visualized in HEK293 transfectants upon chemokine stimulation (0.5µg/ml for 1h). Representative images derived from one out of three independent experiments; scale bar, 25µm. (C) Confocal images of CCR7-YFP2:ZAP70-YFP1 BiFC in transiently transfected MEFs derived from wild-type or β-arrestin 1/2-double deficient mice upon chemokine stimulation (0.5µg/ml for 1h). Representative images derived from one out of three independent experiments; scale bar, 25µm.

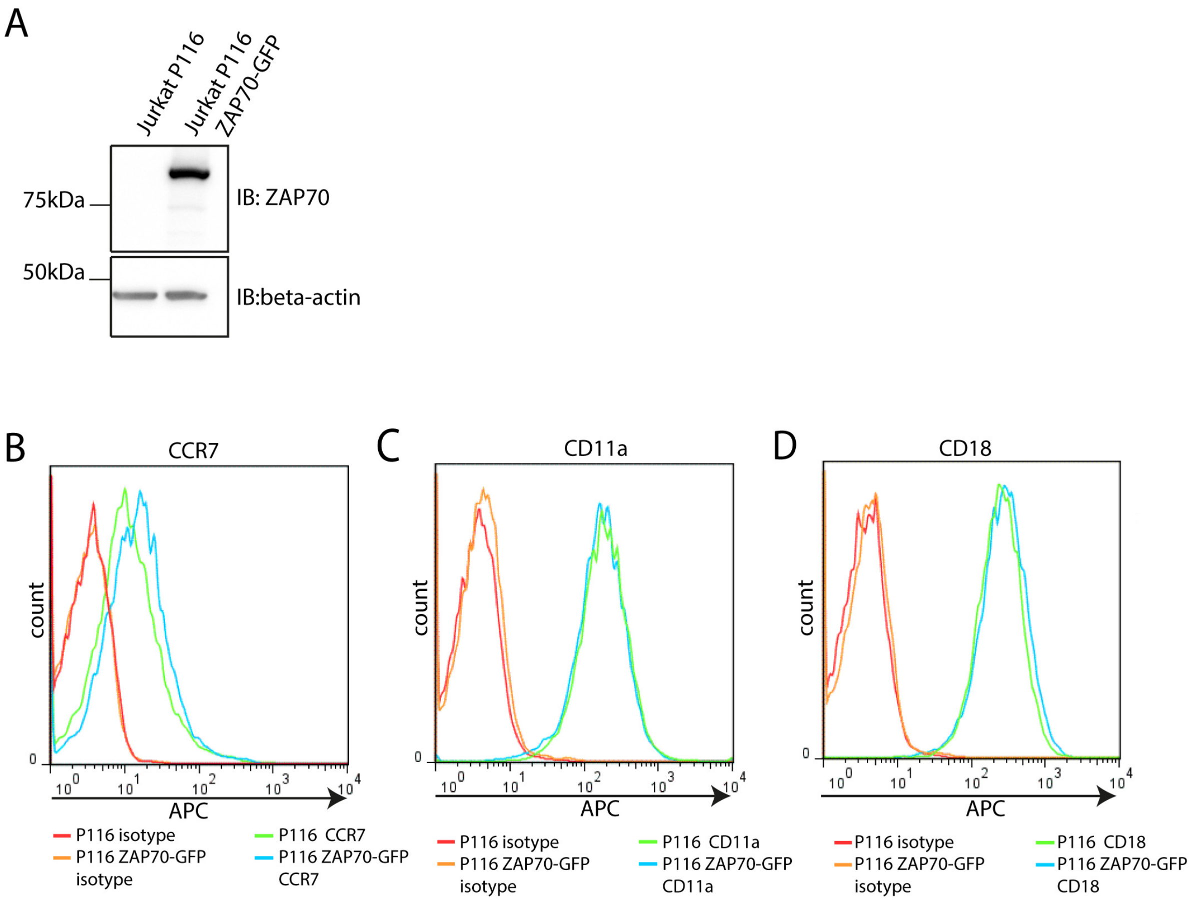

Supplementary Figure 5. Characterization of the Jurkat P116 and Jurkat P116 ZAP70-GFP cell lines. (A) Western blot analysis of lysates derived from Jurkat P116 and Jurkat P116 ZAP70-GFP cells for the expression of ZAP70 and β-actin acting as loading control. One experiment out of three. (B-D) Surface expression of CCR7 (B), CD11a (C) and CD18 (D) on Jurkat P116 and Jurkat P116 ZAP70-GFP cells measured via flow cytometry using specific antibodies.
